# Multimodal Imaging Features of Bilateral Choroidal Ganglioneuroma

**DOI:** 10.1155/2020/6231269

**Published:** 2020-05-11

**Authors:** Zhaoxin Jiang, Ting Zhang, Xing Liu, Dan Liang, Yimin Zhong, Chi-chao Chan, Xiaoyan Ding

**Affiliations:** ^1^State Key Laboratory of Ophthalmology, Zhongshan Ophthalmic Center, Sun Yat-sen University, Guangzhou, China; ^2^Laboratory of Immunology, National Eye Institute, National Institutes of Health, Bethesda, MD, USA

## Abstract

**Purpose:**

Bilateral choroidal ganglioneuroma is extremely rare, and no cases have been described in the literature. Multimodal images are crucial for its diagnosis. Here, we evaluated multimodal images in the early stage of choroidal ganglioneuroma.

**Methods:**

A 6-year-old boy was recruited who had experienced gradually progressive vision loss and rapidly progressive myopia in both eyes over the past 2 years. His eyes were comprehensively evaluated via slit-lamp microscopy, ultrasound biomicroscopy, swept-source optical coherence tomography (SS-OCT), fundoscopy, fundus fluorescein angiography, indocyanine green angiography (ICGA), ultrasound B scanning, and magnetic resonance imaging. Electrophysiological examinations included electrooculography and electroretinography. Choroid biopsy and pathological examination were performed.

**Results:**

Over the past 2 years, the patient's best-corrected visual acuity had gradually decreased to hand motions at 10 cm in the right eye and 20/63 in the left, with axial length growth to 25.89 mm in the right and 28.99 mm in the left. Diffuse thickening in bilateral eyewalls was depicted in B scanning and magnetic resonance imaging. Secondary exudative retinal detachment was evident in SS-OCT and B scanning. SS-OCT depicted low optical reflections in the choroidal layer, revealing a lack of choroidal vasculature. Diffuse hypofluorescence in ICGA photography confirmed extensive loss of choroidal vasculature. In electrophysiological function investigations, electrooculography revealed remarkable bilateral low Arden ratios, with almost extinguished electroretinogram in the right eye. Right-eye choroid biopsy was performed, resulting in a histological diagnosis of choroidal ganglioneuroma.

**Conclusion:**

Choroidal ganglioneuroma is rare. To our knowledge, no bilateral cases have been described in the literature. Major clinical features include a rapid increase in axial length, diffuse choroidal thickening, hyper-reflectivity in the choroid on optical coherence tomography, and loss of choroidal vasculature on ICGA. The current report provides multimodal imaging of choroidal ganglioneuroma for the first time and can be valuable for early diagnosis.

## 1. Introduction

Ganglioneuromas are well-differentiated benign tumors arising from primordial neural crest cells in the sympathetic nervous system [1, 2]. They often present as a solitary, painless, slow-growing mass consisting of ganglion cells, Schwann cells, and fibrous tissue [3]. The most commonly affected sites are the posterior mediastinum (41%), retroperitoneum (37%), adrenal gland (21%), and neck (8%) [4, 5].

Ganglioneuromas have been reported in the orbit, vertebral spines, liver, and lung but are extremely rare in the choroid. A literature search revealed only 13 published case reports [6–18]. In all of those cases, blindness and eye pain were present, and ganglioneuroma was not initially suspected. The choroidal ganglioneuromas were only diagnosed histologically, after ocular enucleation/evisceration. Thus, early suspicion, image analysis, and diagnosis are extremely important in cases of choroidal ganglioneuroma. Only B scanning and magnetic resonance imaging (MRI) features were reported in some of the aforementioned cases, however, and no fundus change examinations including fundus fluorescein angiography (FFA), indocyanine green angiography (ICGA), and optical coherence tomography (OCT) were reported. Herein, we describe a rare pediatric case of a patient with bilateral choroidal ganglioneuroma masquerading as uveitis and explore the multimodal imaging features.

## 2. Patients and Methods

### 2.1. Study Participant

This study was carried out according to the guidelines approved by the Ethics Committee of Zhongshan Ophthalmic Center (ZOC), Sun Yat-sen University, and in accordance with the Declaration of Helsinki. The legal guardian of the patient whose case was described in this report provided written informed consent for its publication.

The patient was a 6-year-old boy who had experienced gradual bilateral loss of vision over the past 2 years. His visual acuity had decreased gradually, and myopia and progressed rapidly; then, he was presented at the Pediatric Ophthalmology Department of the Zhongshan Ophthalmic Center at Sun Yat-sen University in Guangzhou, China. No associated systemic or ocular disease or other obvious predisposing causes were identified. No previous family history of uveitis or neurofibroma was identified. He was diagnosed with uveitis that had developed over the past 2 years. Choroidal abnormality, particularly choroidal tumor, was suspected. Multimodal imaging was performed and evaluated, and choroid biopsy was performed in the right eye.

### 2.2. Multimodal Imaging Acquisition and Analysis

Comprehensive ocular examinations were conducted, including measurement of best-corrected visual acuity (BCVA), intraocular pressure (IOP), and axial length. Slit-lamp microscopy, ultrasound biomicroscopy (UBM), ultrasound B scanning, and MRI were performed. To evaluate the fundus, ultrawidefield scanning laser ophthalmoscopy, spectral domain OCT (SD-OCT), swept-source OCT (SS-OCT), FFA, and ICGA were performed. Electrooculography and electroretinography were performed to evaluate retinal function.

Visual acuity was examined via an Early Treatment Diabetic Retinopathy Study chart (Precision Vision, La Salle, IL, USA). IOP was measured with a noncontact TX-20 Canon tonometer. Anterior segment photographs were obtained using a slit lamp (Haag-Streit, Bern, Switzerland), and UBM was conducted using a model SW-3200L instrument (Tianjin Suowei Electronic Technology Co., Ltd., Tianjin, China). Fundus photography was performed using a Zeiss FF450 instrument (Zeiss, Oberkochen, Germany). OCT images were obtained via spectral domain OCT (Heidelberg Engineering, Heidelberg, Germany). FFA, ICGA, and SD-OCT were performed using a Heidelberg Spectralis HRA (Heidelberg Engineering, Heidelberg, Germany). A-scan ultrasonic biometry (Model Cinescan; Quantel Medical, Clermont-Ferrand, France) was used to measure axial length.

### 2.3. Choroidal Biopsy

Trans-scleral choroidal biopsy was performed as prior studies described [19]. Briefly, biopsy was performed with the patient under hypotensive general anesthesia (systolic blood pressure <80 mmHg). The area of the sclera overlying the lesion to be biopsied was marked, and a half-thickness scleral trapdoor hinged posteriorly was created. Diathermy was applied to the circumference of the proposed biopsy site. An opening of smaller dimensions was then made into the suprachoroidal space within the floor of the lamellar scleral dissection. This opening was enlarged to expose the underlying choroid, which was excised using Vannas' scissors. Care was taken to avoid perforation of the retina. The scleral trapdoor was then sutured to restore the integrity of the globe, and the conjunctiva was closed. The histological stains utilized were hematoxylin/eosin, NeuN, CgA, SOX10, Syn, Phox-2B, S100, HMB-45, and melan-A.

## 3. Results

### 3.1. Clinical Data

Prior to the onset of gradual vision loss and rapid myopia progression in both eyes, the boy's medical history was unremarkable. At first presentation at age 5 years, BCVA was 20/50 in his right eye and 20/25 in his left. Myopia was −2.50 diopter spheres in the right eye and −4.25 spherical equivalent diopter in the left, and respective axial lengths were 25.15 mm and 26.48 mm. At the time of his first visit to the Zhongshan Ophthalmic Center at age 7 years, his BCVA had decreased further to counting fingers at 25 cm in the right eye and 20/63 in the left. His myopia progressed rapidly to −4.00 spherical equivalent diopter in the right eye and −8.25 spherical equivalent diopter in the left, and respective axial lengths were 25.89 mm and 28.99 mm. During a 4-month follow-up period (2 months after biopsy), his BCVA further decreased to hand motions at 10 cm in the right eye and 20/200 in the left, myopia degree was undetectable in the right and −10.00 spherical equivalent diopter in the left, and axial lengths were 25.89 mm in the right and 28.99 mm in the left.

**Figure 1:**
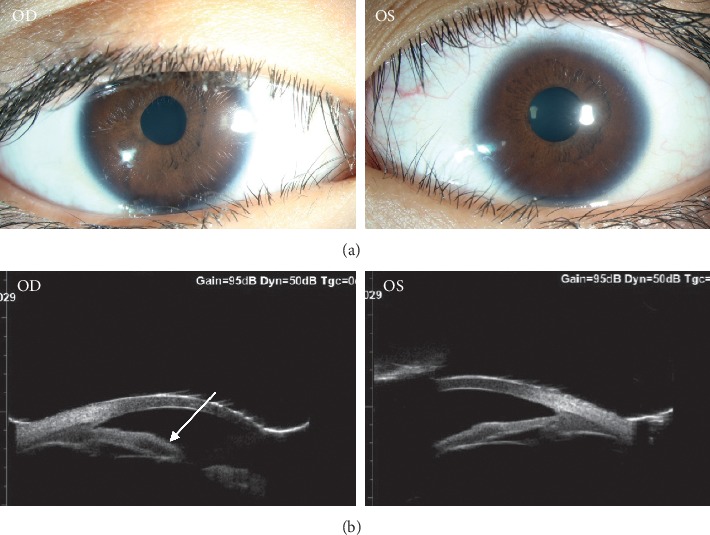
Images of the ocular anterior segment. (a) Bilateral pupils were irregular in shape, a poor light reaction, and no response to any available mydriatic agents. (b) UBM showed remarkable hyporeflection in the iris in right eyes (arrow). Anterior chamber angle was wide-open under the gonioscope. OD, right eye; OS, left eye.

**Figure 2:**
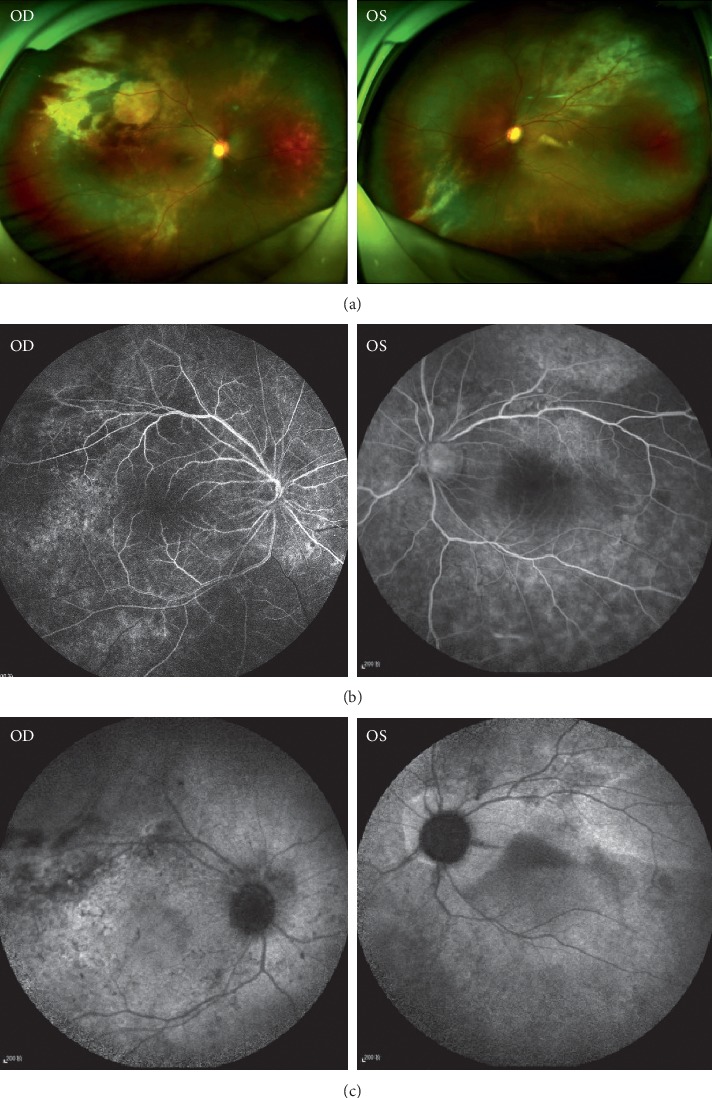
Images of the ocular fundus. (a) SLO showed revealed extensive subretinal hyper-reflective foci in superior-temporal region bilaterally, and exudative retinal detachment was observed in the inferior-temporal region of the retina in SLO in the right eye. (b) FFA showed relatively weak background fluorescence and diffuse mottled RPE, while changes in retinal vasculature were unremarkable. (c) ICGA photography showed diffuse hypofluorescence, revealing the extensive loss of choroidal vasculature.

IOP had fluctuated around the normal range over the past 2 years. An abrupt increased in IOP to 40 mmHg in the left eye was observed on the last visit, but IOP was well controlled with brinzolamide-timolol eyedrops. Corneas and lenses were transparent, but bilateral pupils were irregular in shape, light reactions were poor, and there were no responses to any mydriatic agents. Although IOP was normal, since the initial visit at age of 4 years, his cup-to-disc ratio had become 0.7 : 0.8 bilaterally (Figure 1). Anterior chamber angles were wide-open as determined via gonioscopy, and these observations were confirmed via UBM examination (Figure 2).

### 3.2. Multimodal Imaging of Ganglioneuroma

**Figure 3:**
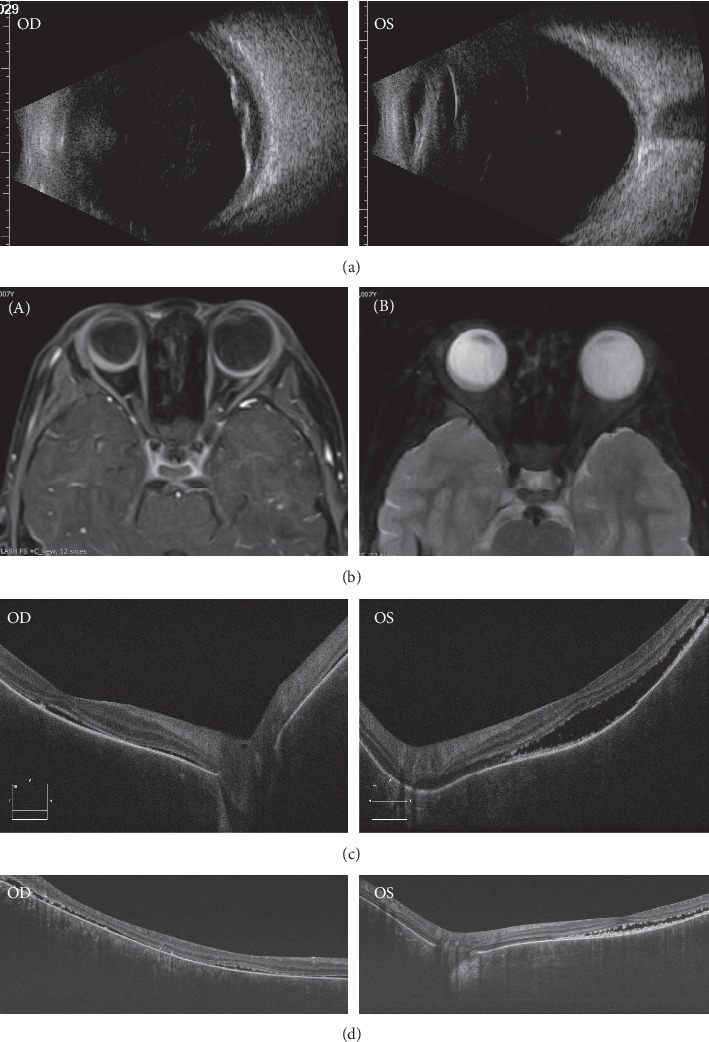
Noninvasive cross section images of the fundus. (a) Ultrasound B scan showed diffuse thickening with the middle strength signal in choroidal layer bilaterally. Secondary retinal detachment was observed in the right eye. (b) MRI showed smooth, diffuse thickening of the eyewall bilaterally: (A) axial, postcontrast fat saturated T1-weighted image; (B) axial, fat saturated T2-weighted image. (c) SD-OCT showed extensive retinal detachment bilaterally. (d) SS-OCT showed low reflections in choroid, revealing the absence of choroidal the vasculature.

UBM, scanning laser ophthalmoscopy, FFA, ICGA, SS-OCT, and MRI were used to assess anatomical structures and functionality. Funduscopy revealed bilateral vasculitis and exudation. Scanning laser ophthalmoscopy depicted exudative retinal detachment in the inferior-temporal region of the retina of the right eye, and this was confirmed via B scanning. SS-OCT revealed extensive subretinal hyper-reflective foci in superior-temporal and macular areas (Figure 3). In evaluations of the vascular system in the retina and choroid, FFA depicted weakened background fluorescence and diffused mottled retinal pigment epithelium (RPE), whereas changes in retinal vasculature were unremarkable (Figure 1). Diffuse ICGA hypofluorescence—and complete lack of fluorescence in some regions—was one of the novel findings accompanying the extensive loss of choroidal vasculature (Figure 1). The choroidal architecture was totally absent in SD-OCT and dramatically obscured in SS-OCT. Lacking choroidal vasculature was noted in SS-OCT (Figure 3(d)), which was taken 1 month after his initial visit (Figure 3(c)). Notably, an absence of the choroidal vasculature was evident (Figure 3). Overlying retinal changes were nonspecific and included retinal atrophy and subretinal fluid that were apparent via OCT. Ultrasound B scanning and MRI depicted bilateral smooth diffuse thickening of the eye walls (Figure 3). These imaging results demonstrated that the choroidal vasculature had been damaged diffusively and severely and was occupied by a nonvascularized solid mass.

### 3.3. Electrophysiological Features of Ganglioneuroma

**Figure 4:**
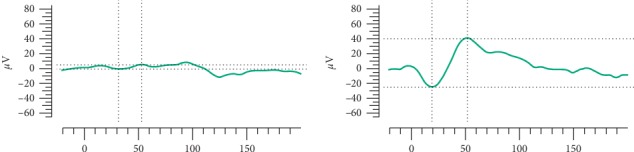
Electrophysiological examination. Almost no respondence was recorded in the right eye, and there was obvious decrease of amplitude in the left eye in ERG.

Electrooculography revealed a remarkably low Arden ratio of 1.1 bilaterally, suggesting severe damage of the RPE and photoreceptors (Figure 4). Electroretinography was unrecordable in the right eye and recordable but markedly abnormal in the left eye.

### 3.4. Histopathological Findings of Ganglioneuroma

**Figure 5:**
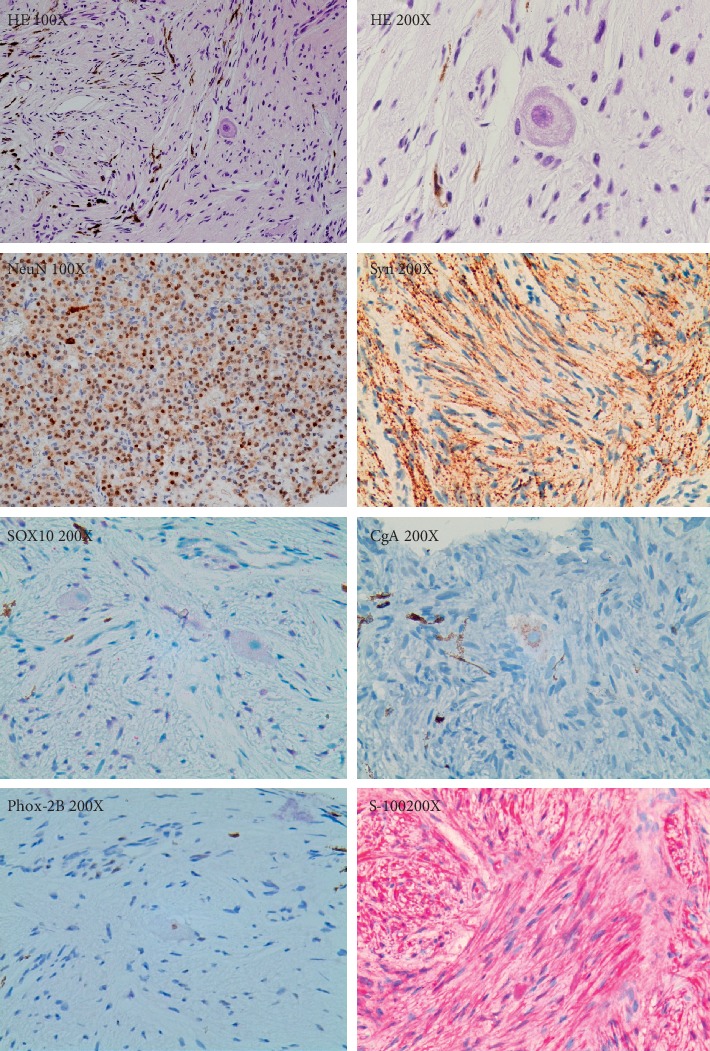
Histopathology of choroid biopsy. Histopathology revealed scattered ganglion and dense infiltrate spindle-shaped cells. Throughout the choroid, ganglion cells demonstrated immunostaining for neuronal markers as NeuN, SOX10, Syn, CgA, and Phox-2B. The spindle-shaped cells were strongly positive for S-100.

Histopathologically, scattered ganglion and dense infiltrating spindle-shaped cells were distributed uniformly throughout the choroid (Figure 5), and neuronal markers including NeuN, CgA, SOX10, Syn, and Phox-2B were positive. The spindle-shaped cells were strongly positive for S-100 but negative for HMB-45 and melan-A. Systemic workup was then performed. No obvious abnormalities were detected via general physical examination, and the results of investigations of the thyroid gland, liver, and adrenal gland were unremarkable.

## 4. Discussion

Choroidal ganglioneuroma is an extremely rare disease, and to date, very limited data pertaining to it have been documented. Due to the difficulty of diagnosis, especially in the early stage, end-stage B scanning and MRI data have been reported in most prior studies. Notably, however, crucial data relating to detailed inspection of the retina and choroid are largely unreported. In the present pediatric patient with rapid bilateral progressive myopia and vision loss, we comprehensively evaluated the anterior and posterior ocular segments. To the best of our knowledge, this is the first case of bilateral choroidal ganglioneuromas to be described in the literature. Multimodal imaging of choroidal ganglioneuromas was performed for the first time, which will provide data that are of early diagnostic value in future cases of putative choroidal ganglioneuroma and facilitate more rapid and informed therapeutic decision-making.

Choroidal ganglioneuromas are highly masquerade according to medical records in this case. First, BCVA was low, and retinal detachment was evident. Thus, the patient's vision loss was initially erroneously primarily attributed to retinal detachment. Second, uveitis had been misdiagnosed because of exudative retinal detachment, extensive subretinal hyper-reflective foci, and mottled RPE. The presence of other conditions including autosomal recessive bestrophinopathy, Coats disease, tuberculosis, and lymphoma had also been considered over the past 2 years. The funduscopy and abnormal Arden ratio make the diagnosis of bestrophinopathy, especially autosomal recessive bestrophinopathy (ARB), very likely. However, a more extensive retinal detachment is characterized in the current case, which is distinguished from Best vitelliform macular dystrophy and ARB. FFA has been helpful in making this disease differentiated from Coats' disease and tuberculous retinopathy, which are characterized by retinal vasculitis and capilliaritis, respectively. The absence of inflammation in the anterior and vitreous cavity, combined with absence of mycobacterial DNA in aqueous humour, may aid in differentiating from intraocular tuberculosis. Ocular lymphoma may be excluded by evaluating the IL-10/IL-6 ratio in aqueous humour.

Multimodal imaging is crucial for the diagnosis of choroidal ganglioneuroma, and the present case yielded several novel and informative observations. Foremost, low optical reflections were depicted in choroidal layers via SS-OCT in accordance with an extensive lack of choroidal vasculature. Diffuse hypofluorescence was depicted in ICGA photography, confirming extensive loss of choroidal vasculature. Smooth and diffuse thickening of the eyewalls was observed via B scanning and MRI, which is consistent with observations in previous studies [7–12]. Lastly, RPE dysfunction was apparent via electrooculography and electroretinography.

Choroidal biopsy is the most reliable method for histological determination of the existence and nature of a choroidal tumor. It entails risks, however, including ocular complications such as intravitreal hemorrhage, retinal detachment, and malignant cell dissemination [20, 21]. When performed by a skillful surgeon, the trans-scleral approach is safe in cases of large lesions that are at a distance from the fovea.

No specific treatment strategy for choroidal ganglioneuroma is advocated in the literature. For ganglioneuroma in retroperitoneums, complete resection is widely considered standard treatment, and prognosis after surgical resection without further therapy is reportedly excellent [22, 23]. Notably, however, resection is not an option in cases of diffuse choroidal ganglioneuroma. Focalized treatment including external radiotherapy may be considered, but ganglioneuroma is not sensitive to radiotherapy or chemical therapy, and ocular complications following radiotherapy are a highly relevant concern [2, 24]. As ganglioneuroma has a tendency to remain asymptomatic for a long time, antiglaucoma treatment and diligent follow-up were utilized in the young boy in the present case.

One limitation of the present study is the deficiency of the case number. Choroidal ganglioneuroma is extremely rare disease that most reported cases were not diagnosed until evisceration/enucleation, and it might be very difficult to recruit enough cases for a case-series study. However, innovative data of multimodal imaging from this case will be valuable for early diagnosis of choroidal ganglioneuroma.

## 5. Conclusion

Choroidal ganglioneuroma is extremely rare, and to our knowledge, no cases of bilateral ganglioneuromas have been previously described. Clinical features include a rapid increase in axial length, diffuse choroidal thickening, choroidal hyper-reflectivity depicted in OCT, and loss of choroidal vasculature depicted in ICGA. Multimodal imaging of choroidal ganglioneuroma is presented herein for the first time, and it is hoped that this will help ophthalmologists to expand their knowledge base with regard to the clinical spectrum of this rare condition and that it may facilitate earlier diagnoses in some future cases.

## Data Availability

All the data used to support the findings of this study are included within the article and are available from the corresponding author by a reasonable request.
